# Emotional Profiles in Adolescents With ADHD: A Person-Centered Analysis of Emotion Regulation, Resilience, and Internalizing Symptoms

**DOI:** 10.31083/AP50136

**Published:** 2026-07-31

**Authors:** Zeynep Seda Albayrak, Mahmoud Mahmoudi, Reyhan Zelal Parlak, Tuba Mutluer, Herdem Aslan Genç, Gülçin Kahraman, Şafak Eray Çamlı, Merve Köksal, Gökçe Koçak Çetin, Ali Evren Tufan, Hale Yapıcı Eser

**Affiliations:** ^1^Department of Child and Adolescent Psychiatry, School of Medicine, Koç University, 34010 Istanbul, Turkey; ^2^Research Center for Translational Medicine (KUTTAM), Koç University, 34010 Istanbul, Turkey; ^3^Department of Psychology, Istanbul Topkapı University, 34087 Istanbul, Turkey; ^4^Department of Child and Adolescent Psychiatry, Faculty of Medicine, Akdeniz University, 07058 Antalya, Turkey; ^5^Department of Child and Adolescent Psychiatry, Faculty of Medicine, Bursa Uludağ University, 16059 Bursa, Turkey; ^6^Department of Child and Adolescent Psychiatry, Faculty of Medicine, Bolu Abant İzzet Baysal University, 14030 Bolu, Turkey; ^7^Department of Psychiatry, Koç University School of Medicine, 34010 Istanbul, Turkey

**Keywords:** attention deficit disorder with hyperactivity, adolescents, self-regulation, psychological resilience, anxiety disorders, depressive disorder

## Abstract

**Background:**

Emotion dysregulation and internalizing symptoms are increasingly recognized as central features of attention-deficit/hyperactivity disorder (ADHD), yet substantial emotional heterogeneity exists among affected adolescents. This study aimed to identify distinct emotional profiles in adolescents with ADHD by integrating emotion regulation difficulties, resilience, and internalizing symptoms using a person-centered, data-driven approach.

**Methods:**

This multicenter, cross-sectional study included 109 clinically referred adolescents aged 11–18 years with a Diagnostic and Statistical Manual of Mental Disorders, Fifth Edition (DSM-5) diagnosis of ADHD. Adolescents completed self-report measures of emotion regulation difficulties (Difficulties in Emotion Regulation Scale–16; DERS-16), resilience (Connor–Davidson Resilience Scale; CD-RISC-25), and internalizing symptoms (Revised Child Anxiety and Depression Scale; RCADS). Parents completed parent-report measures of their child’s ADHD symptom severity (Swanson, Nolan, and Pelham Rating Scale; SNAP-IV) and perceived competence, as well as self-report measures of their own emotion regulation difficulties and resilience. Clinicians rated global severity using the Clinical Global Impression–Severity scale (CGI-S). K-means clustering was applied to standardized adolescent emotional variables to identify distinct profiles, which were evaluated using internal validation indices and clinical correlates. Between-cluster comparisons were conducted across demographic, clinical, and psychosocial variables. Correlation and mediation analyses were performed to examine associations among resilience, emotion regulation difficulties, and internalizing symptoms.

**Results:**

Four distinct emotional profiles were identified: (1) Low Emotion Dysregulation / High Resilience / Low Internalizing, (2) Moderate Emotion Dysregulation / Moderate Resilience / Moderate Internalizing, (3) Low-to-Moderate Emotion Dysregulation / Low Resilience / Low-to-Moderate Internalizing (descriptively labeled as “Low-resilience silent” profile), and (4) High Emotion Dysregulation / Low Resilience / High Internalizing. Emotional profiles differed significantly in emotion regulation difficulties, resilience, and internalizing symptom severity (all *p* < 0.001), but not in parent-reported ADHD symptom dimensions or clinician-rated severity. Perceived social competence, gender distribution, internalizing comorbidity, and medication patterns varied across profiles, whereas parental emotion regulation difficulties and resilience did not. Emotion regulation difficulties showed a significant indirect effect in the association between resilience and internalizing symptoms, such that lower resilience was associated with greater emotion regulation difficulties, which in turn were associated with higher internalizing symptom severity.

**Conclusions:**

Adolescents with ADHD exhibit clinically meaningful emotional profiles that are largely independent of core ADHD symptom severity. Emotion regulation difficulties represent a key mechanism linking resilience and internalizing symptoms. Profile-informed assessment incorporating brief measures of emotion regulation, resilience, and internalizing symptoms may enhance clinical formulation and help identify adolescents with silent emotional vulnerability who may otherwise be overlooked in routine care.

## Main Points

1. Adolescents with attention-deficit/hyperactivity disorder (ADHD) exhibit distinct emotional profiles characterized by different configurations of emotion regulation difficulties, resilience, and internalizing symptoms, independent of core ADHD symptom severity.

2. Emotion regulation difficulties are closely associated with internalizing symptoms and were implicated in a significant indirect association between resilience and anxiety-depressive symptom burden.

3. A clinically relevant subgroup with low resilience and subtle emotional vulnerability (“Low-resilience silent” profile) can be identified despite relatively modest overt symptoms, highlighting the risk of underrecognition in routine clinical practice.

4. Brief self-report measures of emotion regulation and resilience may support profile-informed clinical formulation and help tailor assessment and monitoring beyond traditional ADHD symptom dimensions.

## 1. Introduction

Attention-deficit/hyperactivity disorder (ADHD) is a prevalent neurodevelopmental condition and a major public health concern, affecting approximately 7.6% of children and 5.6% of adolescents worldwide [1]. Although ADHD is traditionally defined by developmentally atypical levels of inattention, hyperactivity, and impulsivity, accumulating evidence indicates that emotional processes constitute a core and clinically consequential dimension of the disorder [2,3]. In particular, emotion dysregulation (ED) has increasingly been conceptualized as a central, transdiagnostic feature of ADHD, contributing substantially to functional impairment across academic, social, and clinical domains [4,5]. Behaviorally, it is reflected in irritability, impulsive emotional outbursts, and difficulties tolerating frustration; affectively, in emotional lability and heightened negative mood; and neurocognitively, in deficits in executive control, error monitoring, and adaptive regulation under stress [3,6]. Meta-analytic evidence indicates that emotional lability in ADHD is a clinically significant correlate of academic underachievement, peer difficulties, and elevated risk for comorbid psychiatric conditions, including mood and substance use disorders [7]. These vulnerabilities may be particularly pronounced during adolescence, a developmental period characterized by delayed cortical maturation, heightened reward sensitivity, and a transition from externally supported to self-directed emotion regulation [8,9]. Together, these processes may increase affective instability and complicate differential diagnosis, particularly with mood and anxiety disorders [10].

Reflecting this multidimensional conceptualization, contemporary dimensional frameworks such as the Research Domain Criteria (RDoC) and the Hierarchical Taxonomy of Psychopathology (HiTOP) position emotion regulation processes at the intersection of behavioral, neural, and developmental levels of ADHD heterogeneity [11]. Consistent with this perspective, person-centered and data-driven approaches—including latent profile analysis, network models, and clustering techniques—have identified distinct patterns of emotional and regulatory functioning within ADHD populations that are not captured by traditional categorical subtypes [2,12]. These findings underscore that ADHD cannot be adequately understood as a unitary cognitive disorder but rather as a heterogeneous condition marked by interacting emotional, cognitive, and neurobiological profiles.

Internalizing symptoms, particularly anxiety and depressive symptoms, represent another clinically important yet frequently underrecognized dimension of ADHD during adolescence [13,14]. Epidemiological and clinical studies suggest that 25%–50% of adolescents with ADHD experience clinically significant anxiety symptoms and that 13%–75% exhibit depressive symptoms, with higher prevalence generally observed among girls [14,15]. Such symptoms are often underdetected due to diagnostic overshadowing by externalizing behaviors and discrepancies between adolescent self-reports and parent or teacher observations [16]. Importantly, emotion dysregulation has been identified as a key mechanism linking ADHD symptoms to internalizing psychopathology, amplifying emotional reactivity and stress sensitivity and thereby increasing vulnerability to anxiety and depression [17]. These emotional difficulties are associated with lower peer acceptance, reduced school engagement, and diminished quality of life [13], supporting the view that emotional symptoms are intrinsic to the ADHD phenotype rather than merely secondary or epiphenomenal [18].

Beyond individual-level neurobiological vulnerability, environmental and psychosocial factors contribute meaningfully to the development and expression of emotion dysregulation [19]. Parenting styles and dimensions [20], exposure to psychosocial adversity [21], and broader contextual stressors shape regulatory development across childhood and adolescence. Within this context, resilience has emerged as a key construct, conceptualized as a dynamic process of positive adaptation that enables individuals to maintain or regain psychological equilibrium in the face of stress [22]. Empirical evidence indicates that resilience and emotion regulation are closely intertwined, with more effective regulatory capacities predicting greater resilience and more adaptive coping [23,24]. Conversely, adolescents with ADHD who exhibit low resilience tend to demonstrate heightened emotional reactivity, increased stress sensitivity, and greater internalizing symptom burden [17].

At the family level, parental emotion regulation and resilience have been theoretically linked to adolescent emotional adjustment through mechanisms of modeling, co-regulation, and the establishment of a supportive emotional climate [25,26]. However, empirical findings are mixed. While some studies report significant maternal or parental effects on adolescent emotional functioning [27], others suggest that these associations attenuate during adolescence as youths develop greater emotional autonomy and increasingly rely on peer-based regulatory processes [28]. This complexity underscores the importance of examining adolescent emotion regulation both within and beyond the family context, particularly during adolescence.

Against this background, the present study adopts a person-centered and exploratory framework to delineate the emotional heterogeneity of adolescents with ADHD by integrating emotion regulation difficulties, resilience, and internalizing symptoms within a person-centered, data-driven framework, without implying developmental trajectories or causal pathways. While the study is conceptually informed by ecological perspectives on adolescent emotional development, the analytic model was operationalized at the adolescent level to preserve conceptual coherence and analytic parsimony. Thus, the ecological framework serves as a theoretical backdrop rather than as a fully integrated multilevel analytic model.

We hypothesized that these emotional constructs would cluster into distinct, clinically meaningful profiles rather than forming a single homogeneous pattern. Specifically, we expected that profiles would differ in internalizing symptom burden, perceived social functioning, gender distribution, and clinical characteristics, while not necessarily reflecting differences in core ADHD symptom severity. To test these hypotheses, we employed a multicenter, cross-sectional design combining adolescent self-reports of emotion regulation, resilience, and internalizing symptoms with parent-reported and clinician-rated measures. In addition, correlation and mediation analyses were conducted to examine whether emotion regulation difficulties demonstrated a significant indirect effect in the association between resilience and internalizing symptoms.

## 2. Materials and Methods

### 2.1 Study Design and Setting

This multicenter, cross-sectional study was conducted between February 2024 and February 2025 at the outpatient child and adolescent psychiatry clinics of Koç University Hospital, Bursa Uludağ University, and Bolu Abant İzzet Baysal University. All assessments were completed during routine outpatient visits. The study aimed to characterize emotional heterogeneity in adolescents with ADHD using a person-centered, data-driven analytic framework.

### 2.2 Participants

Adolescents aged 11–18 years with a clinical diagnosis of ADHD were eligible for inclusion. ADHD diagnoses were established by child and adolescent psychiatrists in accordance with Diagnostic and Statistical Manual of Mental Disorders, Fifth Edition (DSM-5) criteria, based on comprehensive clinical interviews, developmental history, and collateral information obtained from caregivers and school reports.

Exclusion criteria included autism spectrum disorder, intellectual disability, psychotic disorders, major neurological conditions, and chronic medical illnesses likely to substantially affect emotional or cognitive functioning. These criteria were applied to reduce heterogeneity related to neurodevelopmental or medical factors that could confound emotion regulation and internalizing symptom profiles. Other psychiatric comorbidities were not used as exclusion criteria in order to preserve the ecological validity of this clinically referred adolescent ADHD sample.

Each adolescent participated with one parent. A total of 143 adolescents were initially recruited. Following data quality screening and exclusion of incomplete responses, 109 adolescents with complete data on the core clustering variables were retained for the final analytic sample.

### 2.3 Measures

#### 2.3.1 Sociodemographic and Clinical Information

A structured Sociodemographic Data Form was used to collect information on adolescent age and sex, parental age and education, family structure, family psychiatric history, chronic medical conditions, and current psychotropic medication use. Clinical characteristics related to ADHD, including age at diagnosis, were obtained from medical records, whereas clinician-rated severity and current pharmacological treatment were assessed and recorded by the treating psychiatrist during the current clinical visit.

#### 2.3.2 Emotion Regulation Difficulties

Emotion regulation difficulties were assessed using the Difficulties in Emotion Regulation Scale–16 (DERS-16), a 16-item self-report measure developed as a brief version of the original DERS [29,30]. The DERS-16 evaluates five domains of emotion regulation difficulties: nonacceptance of emotional responses, difficulties engaging in goal-directed behavior, impulse control difficulties, lack of emotional clarity, and limited access to emotion regulation strategies. Items are rated on a 5-point Likert scale ranging from 1 (almost never) to 5 (almost always), with higher scores indicating greater emotion regulation difficulties.

In the present study, the DERS-16 was completed by both adolescents (self-report) and their parents (self-report for their own emotion regulation). The Turkish version of the DERS-16 has demonstrated good reliability and construct validity in both adolescent and adult samples, including a stable five-factor structure and satisfactory internal consistency [31,32].

#### 2.3.3 Resilience

Psychological resilience was assessed using the Connor–Davidson Resilience Scale (CD-RISC-25), a 25-item self-report measure originally developed by Connor and Davidson to assess the ability to cope with stress and adversity [33]. Items are rated on a 5-point Likert scale, with higher total scores indicating greater resilience.

In the present study, the CD-RISC-25 was completed by both adolescents and parents as a self-report measure of their own resilience. The Turkish version of the CD-RISC-25, adapted and validated by Karaırmak [34], has demonstrated good reliability and construct validity in adult samples.

#### 2.3.4 Internalizing Symptoms

Internalizing symptoms were assessed using the Revised Child Anxiety and Depression Scale – Child Version (RCADS-CV), a 47-item self-report questionnaire originally developed by Chorpita and colleagues to assess DSM-based anxiety and depressive symptoms in children and adolescents [35]. Items are rated on a 4-point Likert scale ranging from 0 (never) to 3 (always). In the present study, the RCADS-CV Total Internalizing score—reflecting overall anxiety and depressive symptom severity—was used in the primary analyses.

The Turkish version of the RCADS-CV has demonstrated strong internal consistency, a stable six-factor structure, and good convergent and discriminant validity in clinical samples of children and adolescents [36].

#### 2.3.5 Parent-Reported Measures

ADHD symptom severity was assessed using the Swanson, Nolan, and Pelham Rating Scale (SNAP-IV), an 18-item DSM-based checklist corresponding to the DSM-5 inattentive (9 items) and hyperactive–impulsive (9 items) symptom domains. Items are rated on a 4-point Likert scale ranging from 0 (not at all) to 3 (very much), with higher scores indicating greater symptom severity.

In the present study, the SNAP-IV was completed by parents to assess their child’s current ADHD symptoms. The SNAP-IV has been widely used in clinical and epidemiological studies and has demonstrated good psychometric properties in Turkish samples [37].

Parents’ perceptions of their child’s functional competence were assessed using the Perceived Competence Scale–Parent Form (PCS-P), a brief parent-rated measure evaluating the child’s academic competence, social competence, and overall behavioral functioning. Each domain is rated on a 5-point Likert scale, with higher scores reflecting greater perceived competence.

The parent version of the Perceived Competence Scale has been used in Turkish samples to capture functional impairment associated with ADHD symptoms and related psychosocial difficulties [37].

#### 2.3.6 Clinician-Rated Severity

Clinician-rated severity was assessed using the Clinical Global Impression–Severity scale (CGI-S), a widely used one-item clinician-rated measure originally developed for National Institute of Mental Health–sponsored clinical trials [38]. The CGI-S provides a global assessment of overall illness severity based on the clinician’s judgment, integrating symptom burden and functional impairment. Severity is rated on a 7-point scale ranging from 1 (normal, not at all ill) to 7 (among the most extremely ill).

In the present study, the CGI-S was completed by the treating child and adolescent psychiatrist during the clinical visit, based on all available clinical information.

### 2.4 Statistical Analysis

All statistical analyses were conducted using Python version 3.10.0 (Python Software Foundation, Wilmington, DE, USA), the scikit-learn library (open-source Python library; https://scikit-learn.org/), IBM SPSS Statistics version 24.0 (IBM Corp., Armonk, NY, USA), and the PROCESS macro for SPSS (Andrew F. Hayes; https://www.processmacro.org/). Statistical significance was defined as *p* < 0.05 (two-tailed).

#### 2.4.1 Data Preparation

Continuous variables were examined for distributional properties, outliers, and missing data. Clustering analyses were restricted to participants with complete data on the three core clustering variables: emotion regulation difficulties (DERS-16 total score), resilience (CD-RISC-25 total score), and internalizing symptoms (RCADS internalizing total score). Prior to clustering, all variables were standardized using z-scores to ensure comparability across measures [39,40].

#### 2.4.2 Clustering Procedure

A person-centered, data-driven clustering approach was applied to identify distinct emotional profiles among adolescents with ADHD. K-means clustering with *k*-means++ initialization was implemented using the scikit-learn framework [40]. Euclidean distance was used as the similarity metric. Parent-reported variables were not included in the clustering model in order to avoid conflating cross-informant constructs and to limit model dimensionality relative to sample size. Instead, parental variables were examined as correlates in subsequent between-cluster comparisons.

Cluster solutions were systematically explored across a range of cluster numbers (*k* = 2–12), consistent with prior psychiatric clustering applications employing symptom-scale data [41,42]. To reduce sensitivity to random initialization, clustering was repeated across 100 independent runs for each k (n_init = 100) [43].

To further evaluate the robustness of the clustering solution, additional stability analyses were conducted. Sensitivity to random initialization was assessed by repeating k-means clustering across multiple runs and quantifying agreement between resulting partitions using pairwise Jaccard similarity coefficients. Convergence stability under extensive optimization was examined using an increased number of internal initializations (n_init = 100). Robustness to sampling variability was further evaluated using bootstrap resampling, with agreement between bootstrap-derived partitions and the original clustering solution quantified using the Adjusted Rand Index (ARI). These procedures were implemented to assess the reproducibility of the clustering structure within the present sample.

#### 2.4.3 Model Evaluation and Selection

Candidate cluster solutions were evaluated using complementary validation metrics capturing cluster compactness, separation, and separability. These included the Silhouette coefficient [44], the Davies–Bouldin Index (DBI) [45], and Linear Discriminant Analysis (LDA)–based separability accuracy treating cluster assignments as pseudo-labels [42,46]. LDA was used as a diagnostic tool to quantify the degree to which the derived clusters could be linearly separated in multivariate space, rather than as a supervised classification model.

Because no single index provides a definitive criterion for optimal clustering, validation metrics were interpreted jointly, and a composite ranking score was used to summarize performance across runs. The composite score combined normalized DBI, Silhouette, and LDA accuracy metrics together with their across-run stability, quantified as the standard deviation (SD) of each metric across repeated runs. In parallel, *k*-means inertia (within-cluster sum of squared distances) was examined using the elbow method to identify regions of diminishing returns with increasing *k* [40].

Final model selection balanced statistical support with clinical interpretability, favoring solutions that yielded distinct and clinically meaningful profiles.

#### 2.4.4 Between-Cluster Comparisons

Following identification of the final cluster solution, clusters were compared on demographic variables, clinical characteristics, ADHD symptom dimensions (Inattention and Hyperactivity–Impulsivity), perceived competence, parent-reported psychosocial variables (parent emotion regulation difficulties and parent resilience), and medication use. Due to non-normal distributions, continuous variables were analyzed using Kruskal–Wallis tests, with post hoc pairwise comparisons performed using Dunn’s test with Bonferroni correction when overall group differences were significant. Categorical variables were compared using chi-square tests.

#### 2.4.5 Correlation Analyses

Bivariate associations among emotion regulation difficulties, resilience, and internalizing symptoms were examined using Pearson or Spearman correlation coefficients, as appropriate based on variable distributions.

#### 2.4.6 Mediation Analysis

To examine whether emotion regulation difficulties statistically mediated the association between adolescent resilience and internalizing symptom severity, a mediation analysis was conducted using the PROCESS macro for SPSS (Model 4). Adolescent resilience (CD-RISC-25 total score) was specified as the independent variable, emotion regulation difficulties (DERS-16 total score) as the mediator, and internalizing symptoms (RCADS internalizing total score) as the dependent variable.

Indirect effects were estimated using bias-corrected bootstrap procedures with 5000 resamples. An indirect effect was considered statistically significant if the 95% bootstrap confidence interval did not include zero. Direct, indirect, and total effects were reported to characterize partial versus full mediation patterns. Given the cross-sectional design, mediation analyses were interpreted as a statistical decomposition of associations rather than as evidence of temporal or causal relationships.

## 3. Results

### 3.1 Sample Characteristics

Of the initially recruited adolescents (N = 143), 109 participants with complete data on the core clustering variables were retained for analysis. The final analytic sample comprised 109 adolescents diagnosed with ADHD, of whom 48.6% were female. The mean age was 13.8 ± 2.0 years (range: 11–18). Mothers’ and fathers’ mean ages were 42.9 ± 6.0 and 46.3 ± 5.7 years, respectively. Most adolescents lived with both parents (77.1%), while 17.4% had divorced parents and 5.5% reported other family arrangements.

Clinician-rated CGI-S scores indicated a moderate level of ADHD severity (mean = 4.8 ± 1.5), overall. According to the parental report, perceived competence total score was 10.5 ± 2.2 (range: 3–15). Internalizing psychiatric comorbidities were present in 23.9% of the sample, neurodevelopmental comorbidities in 11.9%, and externalizing disorders in 8.3%. A family history of psychiatric disorder was reported in 27.5% of the cases.

Regarding pharmacological treatment, 67.9% of the adolescents were receiving methylphenidate, 12.8% atomoxetine, 9.2% selective serotonin reuptake inhibitors (SSRIs), and 10.1% antipsychotic medication; one participant was treated with guanfacine. Detailed demographic and clinical characteristics are presented in Table 1.

**Table 1.** **Sociodemographic and clinical characteristics of the study sample (n = 109)**.

Variable	Value
Age, years	13.8 ± 2.0
Maternal age, years	42.9 ± 6.0
Paternal age, years	46.3 ± 5.7
Sex – Female, n (%)	53 (48.6)
Sex – Male, n (%)	56 (51.4)
Family psychiatric history, n (%)	30 (27.5)
Family chronic physical illness, n (%)	26 (23.9)
DERS total score	41.3 ± 14.2
CD-RISC total score	55.2 ± 16.4
RCADS internalizing total	53.4 ± 25.7
CGI-S score	4.8 ± 1.5
Perceived competence total	10.5 ± 2.2
Methylphenidate use, n (%)	74 (67.9)
Atomoxetine use, n (%)	14 (12.8)
SSRI use, n (%)	10 (9.2)
Antipsychotic use, n (%)	11 (10.1)
Guanfacine use, n (%)	1 (0.9)
Comorbid internalizing disorder, n (%)	26 (23.9)
Comorbid neurodevelopmental disorder, n (%)	13 (11.9)
Comorbid externalizing disorder, n (%)	9 (8.3)

Values are presented as mean ± SD or n (%). SD, standard deviation; DERS, Difficulties in Emotion Regulation Scale; CD-RISC, Connor–Davidson Resilience Scale; RCADS, Revised Child Anxiety and Depression Scale; CGI-S, Clinical Global Impression–Severity scale; SSRI, selective serotonin reuptake inhibitor. Percentages for medication use may exceed 100% due to concurrent treatments.

### 3.2 Evaluation of Candidate Cluster Solutions

**Fig. 1.**
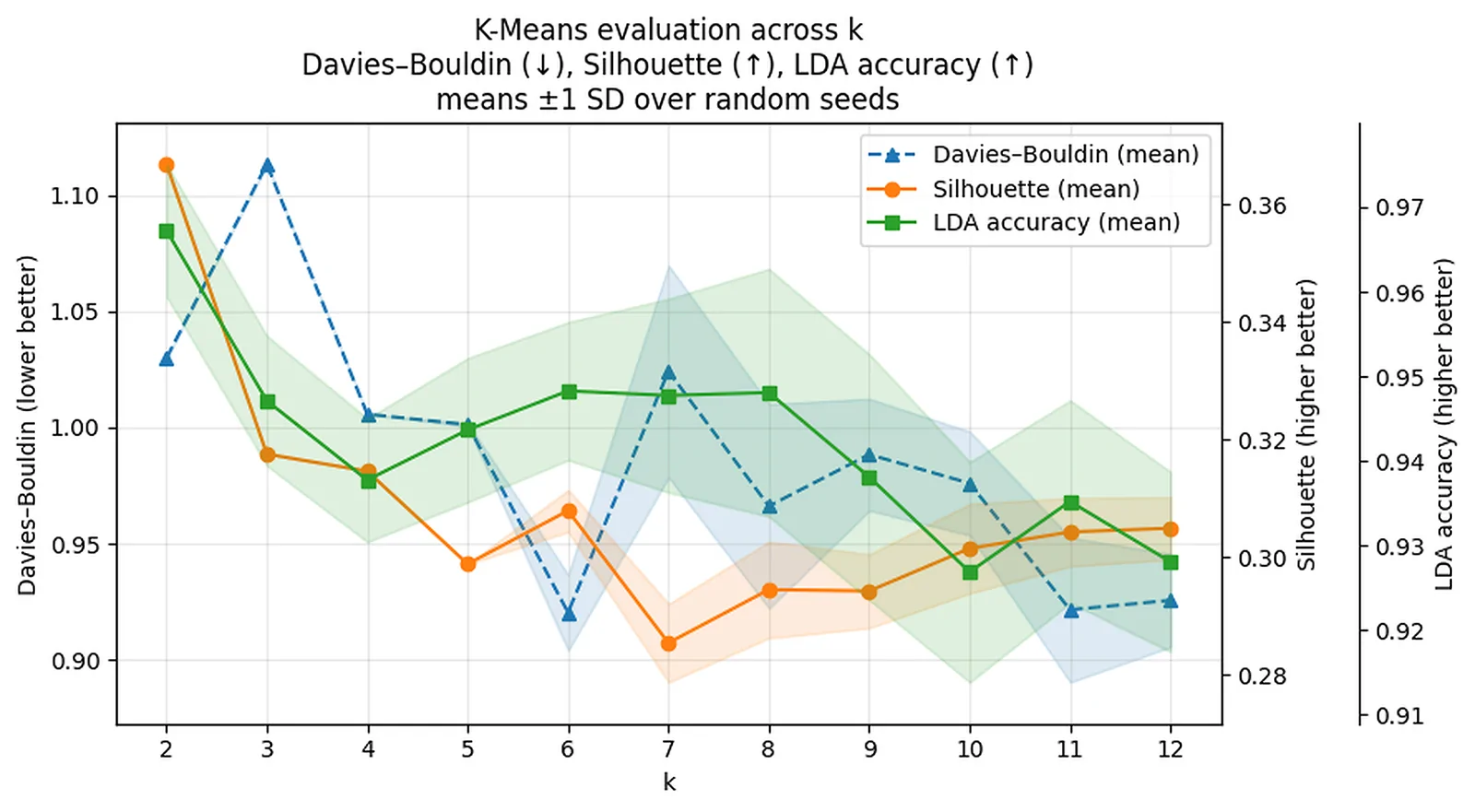
**Evaluation of clustering solutions across values of *k***. Mean Davies–Bouldin Index (DBI), Silhouette coefficient, and Linear Discriminant Analysis (LDA) classification accuracy across cluster solutions ranging from *k* = 2 to *k* = 12. Values represent means ± SD across multiple random initializations. Lower DBI values indicate better cluster compactness and separation, whereas higher Silhouette coefficients and LDA accuracy indicate improved clustering quality and separability. Arrows indicate the direction of better performance for each metric: lower values are better for the Davies–Bouldin index, whereas higher values are better for silhouette and LDA accuracy.

Clustering solutions were systematically evaluated across values of *k* ranging from 2 to 12 using complementary internal clustering quality, stability, and diagnostic separability indices. Mean DBI values, Silhouette coefficients, and LDA–based separability accuracy across random initializations are shown in Fig. 1. Across the tested range, no single value of *k* emerged as uniformly optimal across all indices. Instead, several mid-range solutions demonstrated comparable quantitative performance, indicating multiple near-equivalent clustering structures.

**Fig. 2.**
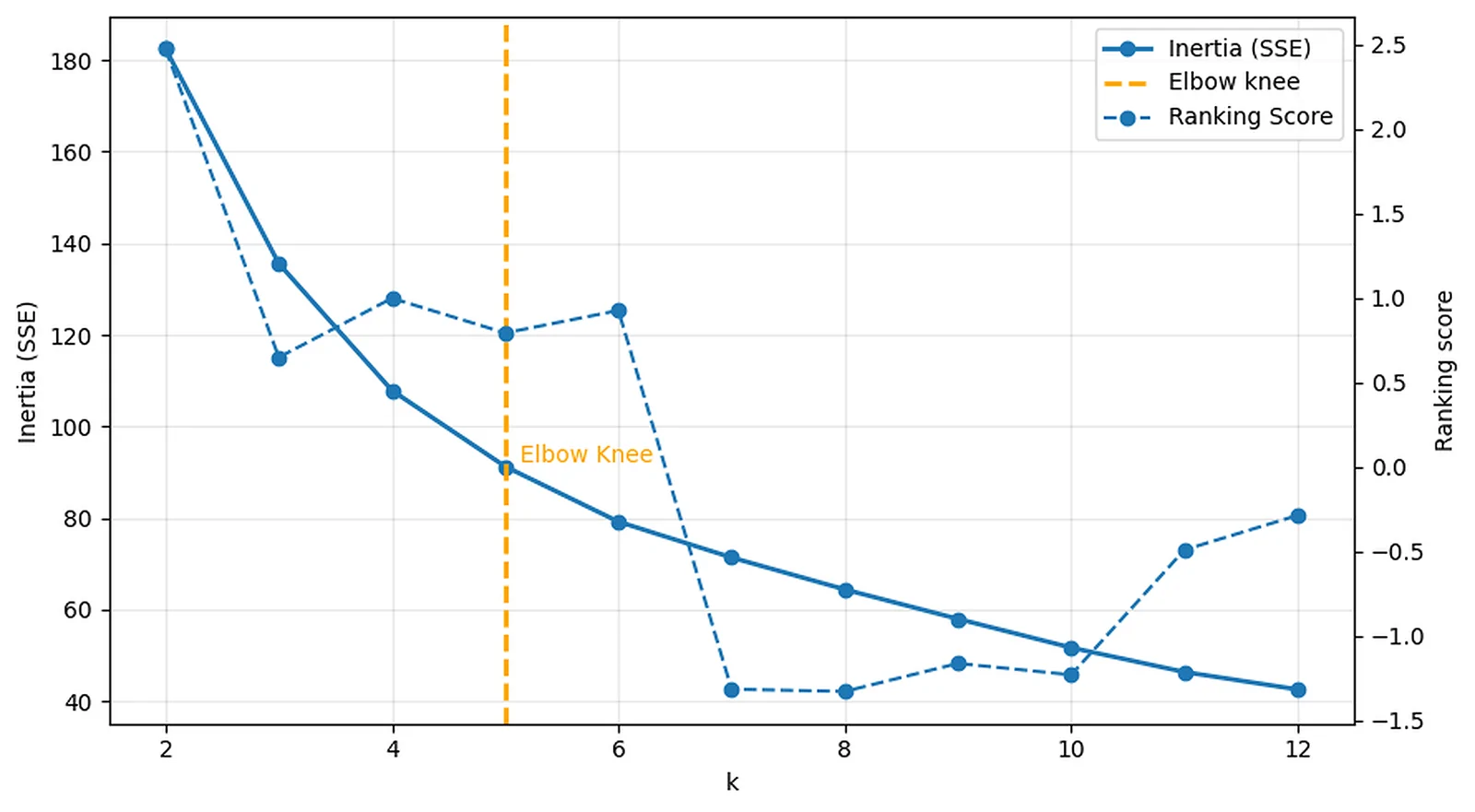
**Inertia and composite ranking score across candidate cluster solutions**. K-means inertia (sum of squared errors, SSE) and composite ranking scores plotted across values of *k*. The dashed vertical line indicates the elbow region identified by inspection of the inertia curve. Composite ranking scores integrate normalized clustering performance and stability metrics across repeated runs, with higher scores reflecting more favorable solutions.

To integrate clustering quality and robustness, a composite ranking score combining normalized performance and stability metrics was computed for each candidate solution and examined alongside k-means inertia using the elbow method (Fig. 2). The inertia curve showed a clear reduction in slope around k ≈ 5, suggesting a region of diminishing returns. The composite ranking consistently identified k = 4 and k = 6 among the highest-ranking non-trivial solutions.

### 3.3 Selection of the Final Cluster Solution

**Fig. 3.**
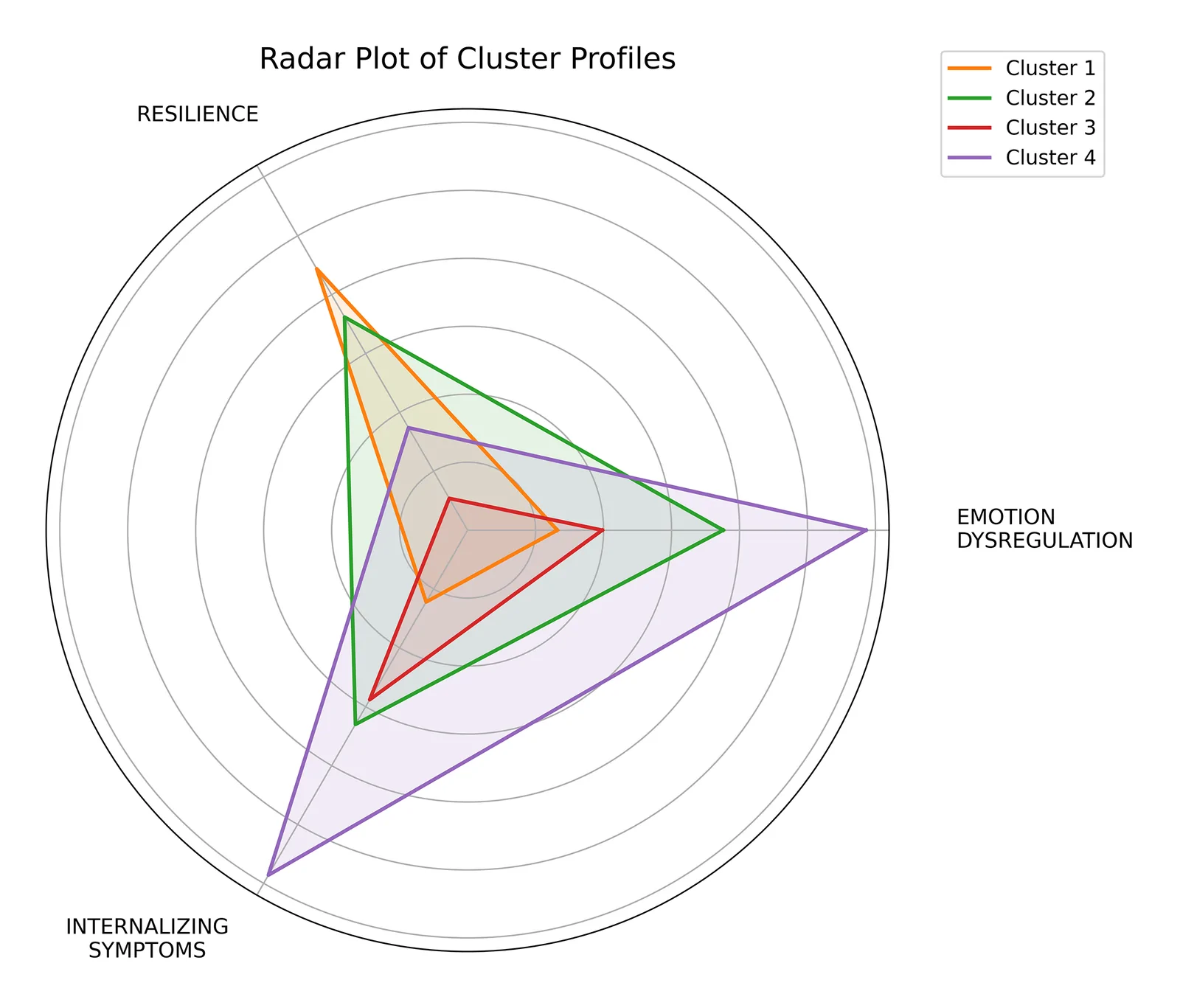
**Radar plot of four-cluster emotional profiles**. Radar plot depicting standardized mean scores (z-scores) of emotion regulation difficulties (DERS-16 total), resilience (CD-RISC-25 total), and internalizing symptoms (RCADS internalizing total) for the four identified emotional profiles: Low Emotion Dysregulation / High Resilience / Low Internalizing, Moderate Emotion Dysregulation / Moderate Resilience / Moderate Internalizing, Low-to-Moderate Emotion Dysregulation / Low Resilience / Low-to-Moderate Internalizing, and High Emotion Dysregulation / Low Resilience / High Internalizing.

Given the comparable quantitative support for *k* = 4 and *k* = 6, final model selection was guided by interpretability and parsimony. Visual inspection of standardized cluster profiles revealed that the four-cluster solution yielded distinct, non-overlapping configurations across emotion regulation difficulties, resilience, and internalizing symptoms (Fig. 3). In contrast, the six-cluster solution introduced profiles with highly similar geometries that differed primarily in magnitude rather than pattern, suggesting increased fragmentation without corresponding gains in conceptual clarity (see **Supplementary Fig. 1**). Accordingly, the four-cluster solution was selected as the final model.

Stability analyses indicated consistent convergence of the four-cluster solution across repeated optimization procedures. Mean pairwise Jaccard similarity for the four-cluster solution was 0.63 under single-initialization conditions and increased to 0.97 under extensive optimization (n_init = 100). Bootstrap resampling further demonstrated reproducible structure under sampling perturbation (mean ARI = 0.72). Detailed stability metrics and visualization of clustering behavior across candidate solutions are provided in **Supplementary Figs. 2,3**.

### 3.4 Description of Emotional Profiles

The four identified emotional profiles were characterized as follows:

1. Low Emotion Dysregulation / High Resilience / Low Internalizing Profile: Adolescents in this cluster exhibited low levels of emotion regulation difficulties, high resilience, and minimal internalizing symptoms, reflecting an emotionally regulated and adaptive pattern.

2. Moderate Emotion Dysregulation / Moderate Resilience / Moderate Internalizing Profile: This cluster was characterized by intermediate levels of dysregulation, resilience, and internalizing symptoms, representing a mildly at-risk emotional pattern.

3. Low-to-Moderate Emotion Dysregulation / Low Resilience / Low-to-Moderate Internalizing Profile (hereafter referred to as the descriptively labeled as “Low-resilience silent” profile): Despite relatively modest emotion regulation difficulties, adolescents in this cluster displayed markedly reduced resilience accompanied by low-to-moderate internalizing symptoms, consistent with a pattern of relative or “silent” vulnerability.

4. High Emotion Dysregulation / Low Resilience / High Internalizing Profile: Adolescents in this cluster demonstrated pronounced emotion regulation difficulties, low resilience, and high internalizing symptom severity, representing the most emotionally vulnerable group (Fig. 3).

### 3.5 Between-Cluster Comparisons

#### 3.5.1 Demographic Variables

No significant differences were observed among clusters with respect to adolescent age, maternal age, paternal age, or parental education levels (all *p* > 0.05).

#### 3.5.2 Core Emotional Constructs

Significant between-cluster differences were observed for all three clustering variables. Kruskal–Wallis tests revealed robust differences in emotion regulation difficulties (DERS-16 total: H = 79.4, *p* < 0.001), resilience (CD-RISC-25 total: H = 61.6, *p* < 0.001), and internalizing symptoms (RCADS internalizing total: H = 74.8, *p* < 0.001).

Post hoc analyses showed that the Low Emotion Dysregulation / High Resilience / Low Internalizing profile differed significantly from all other clusters on each core construct. The High Emotion Dysregulation / Low Resilience / High Internalizing profile exhibited the highest dysregulation and internalizing symptom burden and the lowest resilience. The two intermediate profiles demonstrated overlapping but distinct patterns, with the Low-to-Moderate Emotion Dysregulation / Low Resilience / Low-to-Moderate Internalizing Profile primarily differentiated by reduced resilience rather than elevated symptom severity.

Between-cluster differences in emotion regulation difficulties, resilience, and internalizing symptom severity are presented in Table 2.

**Table 2.** **Emotion regulation, resilience, and internalizing symptoms across emotional clusters**.

Variable	Cluster 1 (n = 39)	Cluster 2 (n = 29)	Cluster 3 (n = 19)	Cluster 4 (n = 22)	*p*
DERS total score	29.5 ± 6.7	46.6 ± 7.4	34.1 ± 7.9	61.5 ± 7.1	<0.001
Resilience (CD-RISC) total score	66.9 ± 11.7	60.3 ± 8.8	35.2 ± 7.1	44.9 ± 14.8	<0.001
RCADS internalizing symptoms total	30.5 ± 11.9	57.3 ± 12.8	51.9 ± 19.9	90.0 ± 13.5	<0.001
Perceived social competence	3.92 ± 0.93	3.54 ± 0.79	3.11 ± 1.05	3.82 ± 1.05	0.018

Values are presented as mean ± SD. Group comparisons were conducted using the Kruskal–Wallis test.

Detailed subscale-level comparisons of emotion regulation and internalizing symptoms across clusters are provided in **Supplementary Table 1**.

#### 3.5.3 ADHD Symptom Dimensions, Clinical Severity and Parental Measures

Clusters did not differ significantly in parent-reported Inattention or Hyperactivity–Impulsivity scores, clinician-rated CGI-S scores, or age at ADHD diagnosis (all *p* > 0.05), indicating that emotional profiles were largely independent of core ADHD symptom severity and clinician-rated global severity.

Similarly, parental self-reported emotion regulation difficulties and resilience did not differ significantly across clusters (parental DERS: H = 1.099, *p* = 0.577; parental resilience: H = 2.858, *p* = 0.240).

#### 3.5.4 Perceived Competence

Perceived social competence differed significantly across clusters (H = 10.0, *p* = 0.018). Post hoc analyses indicated that parents of adolescents in the Low-to-Moderate Emotion Dysregulation / Low Resilience / Low-to-Moderate Internalizing (Low-resilience silent) profile reported significantly lower social competence compared with those in the Low Emotion Dysregulation / High Resilience / Low Internalizing profile. No significant differences in perceived social competence were observed among the remaining profiles. Academic and behavioral competence did not differ significantly across clusters.

#### 3.5.5 Sex, Medication Use, and Comorbidity

Sex distribution differed significantly across clusters (χ^2^ = 13.7, *p* = 0.003), with female adolescents overrepresented in the High Emotion Dysregulation / Low Resilience / High Internalizing profile.

Medication patterns also differed significantly across clusters. The frequency of methylphenidate use was higher in the Low Emotion Dysregulation / High Resilience profile and lower in the High Emotion Dysregulation / Low Resilience / High Internalizing profile (χ^2^ = 8.9, *p* = 0.031). In contrast, SSRI use was most frequent in the High Emotion Dysregulation / Low Resilience / High Internalizing profile (χ^2^ = 14.7, *p* = 0.002). No significant differences were observed in atomoxetine or antipsychotic use.

The prevalence of internalizing psychiatric comorbidities differed significantly across clusters (χ^2^ = 18.3, *p* < 0.001), with the highest rates observed in the High Emotion Dysregulation / Low Resilience / High Internalizing profile. Other comorbidity categories and family history variables did not differ significantly across clusters. A summary of between-cluster comparisons is presented in Table 3.

**Table 3.** **Clinical and treatment-related characteristics across emotional clusters**.

Variable	Cluster 1 n (%)	Cluster 2 n (%)	Cluster 3 n (%)	Cluster 4 n (%)	*p*
Female sex	14 (35.9)	10 (34.5)	12 (63.2)	17 (77.3)	0.003
Methylphenidate use	32 (82.1)	20 (69.0)	12 (63.2)	10 (45.5)	0.031
SSRI use	0 (0.0)	1 (3.4)	3 (15.8)	6 (27.3)	0.002
Comorbid internalizing disorder	3 (7.7)	5 (17.2)	6 (31.6)	12 (54.5)	<0.001

Values are presented as n (%). Group comparisons were performed using chi-square or Fisher’s exact tests, as appropriate.

### 3.6 Correlation and Mediation Analyses

Across the full sample, greater emotion regulation difficulties were significantly associated with lower resilience and higher internalizing symptom severity, while resilience was inversely associated with internalizing symptoms (all *p* < 0.001).

### 3.7 Mediation Analysis

To further examine the relational structure among resilience, emotion regulation difficulties, and internalizing symptoms, a mediation analysis was conducted across the full sample. Emotion regulation difficulties demonstrated a significant indirect effect in the association between adolescent resilience and internalizing symptom severity.

Higher resilience was significantly associated with lower levels of emotion regulation difficulties (path a: b = –0.26, standard error [SE] = 0.08, *t* = –3.32, *p* = 0.001), accounting for 9.4% of the variance in DERS-16 total scores. When both resilience and emotion regulation difficulties were entered simultaneously into the model predicting internalizing symptoms, emotion regulation difficulties emerged as a strong positive predictor (path b: b = 1.20, SE = 0.13, *t* = 9.58, *p* < 0.001). Resilience remained a significant negative predictor after accounting for emotion regulation difficulties (direct effect c′: b = –0.28, SE = 0.11, *t* = –2.61, *p* = 0.010). The full model explained 54.2% of the variance in internalizing symptoms.

The total effect of resilience on internalizing symptoms was significant (path c: b = –0.60, SE = 0.14, *t* = –4.28, *p* < 0.001). The indirect effect of resilience on internalizing symptoms through emotion regulation difficulties was also significant (b = –0.32), with a bias-corrected 95% bootstrap confidence interval that did not include zero (BootSE = 0.11, 95% confidence interval (CI) [–0.54, –0.11]), indicating partial mediation.

**Fig. 4.**
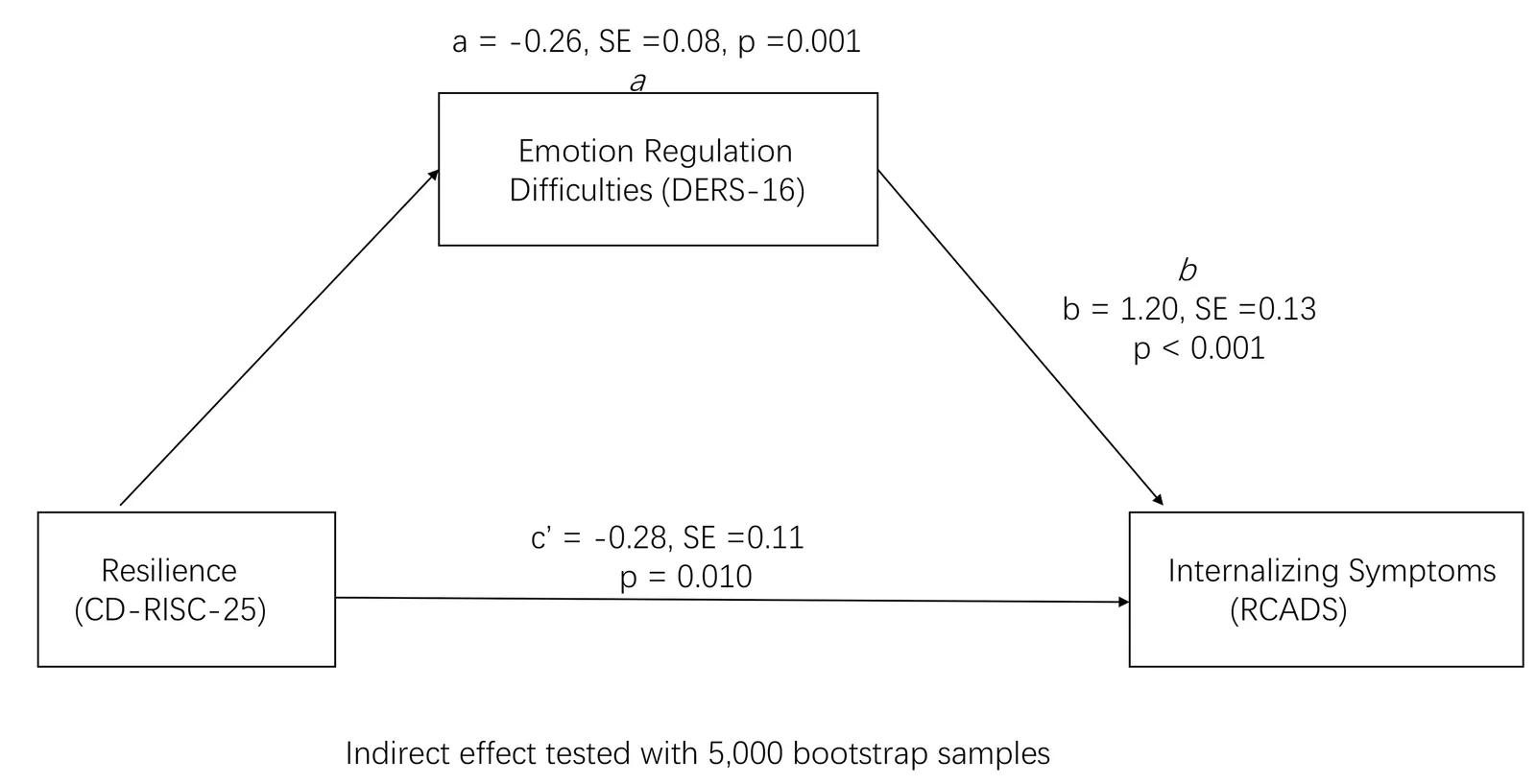
**Mediation model of resilience, emotion regulation difficulties, and internalizing symptoms**. Schematic representation of the mediation model examining the indirect association between adolescent resilience and internalizing symptoms through emotion regulation difficulties. Unstandardized regression coefficients are displayed for each path. The indirect effect was estimated using bias-corrected bootstrap procedures with 5000 resamples. *p* < 0.05 indicates statistical significance. The letters a, b, and c′ denote standard mediation paths: a = resilience to emotion regulation difficulties, b = emotion regulation difficulties to internalizing symptoms, controlling for resilience, and c′ = direct effect of resilience on internalizing symptoms, controlling for emotion regulation difficulties. SE, standard error.

A schematic representation of the mediation model is presented in Fig. 4, and detailed path coefficients are provided in **Supplementary Table 2**.

## 4. Discussion

The present study yielded four distinct emotional profiles based on combinations of emotion regulation difficulties, resilience, and internalizing symptoms. These profiles differed in emotional constructs, perceived social competence, gender distribution, internalizing comorbidity, and medication patterns, while not differing in core ADHD symptom severity or clinician-rated global severity. In addition, emotion regulation difficulties demonstrated a significant indirect association between resilience and internalizing symptoms.

Taken together, these findings suggest that emotional heterogeneity in adolescent ADHD extends beyond variation in core symptom severity and may be better understood in terms of regulatory capacity and internalizing vulnerability. The following discussion interprets these patterns within existing theoretical frameworks and considers their clinical implications, while acknowledging the exploratory and cross-sectional nature of the analyses.

Consistent with prior person-centered studies in ADHD and related populations [2,12], our findings demonstrate distinct emotional profiles that are not captured by symptom counts or traditional categorical subtypes. Profiles ranged from a resilient, emotionally regulated group with minimal internalizing symptoms to a highly vulnerable group marked by pronounced emotion dysregulation, low resilience, and elevated anxiety and depressive symptoms. Importantly, intermediate profiles reflect a configuration characterized by a relative imbalance between resilience and symptom burden rather than a simple gradation of severity. This clinical interpretability also informed our preference for the four-cluster solution over the six-cluster alternative, which primarily subdivided intermediate profiles by magnitude without yielding clinically distinct patterns. Notably, the Low-to-Moderate Emotion Dysregulation / Low Resilience / Low-to-Moderate Internalizing profile demonstrated reduced adaptive capacity despite relatively modest dysregulation and symptom levels, suggesting a unique vulnerability pattern. These results align with dimensional frameworks such as RDoC and HiTOP [5,24,47], supporting the conceptualization of ADHD as a heterogeneous condition comprising multiple emotional phenotypes.

### 4.1 Internalizing Symptoms and Emotion Regulation as Central Mechanisms

Internalizing symptoms were strongly associated with emotion regulation difficulties across the sample and varied substantially across emotional profiles. Adolescents in the High Emotion Dysregulation / Low Resilience / High Internalizing profile exhibited the greatest internalizing burden, whereas emotionally regulated profiles showed minimal anxiety and depressive symptoms despite comparable ADHD severity. This pattern supports the view that internalizing psychopathology in ADHD is closely linked to shared regulatory vulnerabilities rather than representing a nonspecific consequence of symptom severity or global impairment [48,49,50].

Mediation analyses further indicated that emotion regulation difficulties demonstrated a significant indirect effect in the association between resilience and internalizing symptoms [51]. Higher resilience was associated with fewer emotion regulation difficulties, which in turn were associated with lower internalizing symptom severity, with a residual direct association between resilience and internalizing symptoms also persisting [52]. Although causal inferences cannot be drawn from the cross-sectional design, this relational structure is consistent with theoretical models positing emotion regulation as a key pathway through which adaptive capacities may buffer against internalizing risk in ADHD and other neurodevelopmental conditions [17,24,53]. From a clinical perspective, distinguishing between shared and residual components of the association may help clarify whether assessment and intervention efforts should prioritize emotion regulation processes, resilience-enhancing strategies, or both, rather than treating these constructs as interchangeable indicators of internalizing risk. In this context, reliance solely on overt symptom severity may overlook adolescents whose adaptive capacity is limited despite relatively modest clinical presentations [21,24].

Importantly, beyond confirming that resilience, emotion regulation difficulties, and internalizing symptoms are interrelated, the mediation model provides a more structured characterization of their relational organization. By partitioning the total association into indirect and residual direct components, it illustrates that emotion regulation difficulties represent a statistically differentiated component of this association rather than merely a redundant correlate. Although temporal or mechanistic inferences cannot be drawn from cross-sectional data, this analytic framing offers a more nuanced understanding of how adaptive capacity and emotional distress co-occur in adolescents with ADHD.

### 4.2 Clinical Relevance of the Low-resilience Silent Profile

A clinically relevant observation of the present study is the identification of a Low-to-Moderate Emotion Dysregulation / Low Resilience / Low-to-Moderate Internalizing profile (Low-resilience silent). Adolescents in this cluster did not present with prominent internalizing symptoms or marked emotional lability, yet exhibited substantially reduced resilience and lower perceived social competence. This pattern suggests that reliance solely on overt symptom severity may overlook adolescents whose adaptive coping resources are limited but whose distress is not expressed through clinically salient complaints [2,5,54].

Importantly, this profile is not readily explained as a milder severity variant of the high-vulnerability group, nor simply an intermediate position along a linear severity continuum [2,12]. In contrast to the Low Emotion Dysregulation / High Resilience / Low Internalizing profile, adolescents in the Low-resilience silent configuration exhibited substantially reduced resilience despite only modest levels of dysregulation and internalizing symptoms. This relative imbalance between adaptive capacity and symptom burden differentiates the profile conceptually: resilience appears disproportionately low in relation to observable emotional symptoms [22,23]. Clinically, such a pattern may reflect constrained coping resources that are not yet accompanied by overt psychopathology but may confer vulnerability under increasing developmental or contextual demands [21,24].

This finding reinforces contemporary resilience frameworks emphasizing that resilience is not merely the inverse of psychopathology but rather a distinct and dynamic dimension of adaptation with functional significance [22,23]. In the context of ADHD, where developmental trajectories are highly heterogeneous, diminished resilience may represent a latent vulnerability that becomes clinically salient under increasing academic, social, or emotional demands [12,55]. Person-centered profiling approaches that capture such silent vulnerabilities may therefore have important implications for early identification and preventive intervention [54,56].

From a clinical standpoint, this profile may be suspected when adolescents report low coping confidence or demonstrate reduced flexibility under stress despite only mild anxiety or depressive symptom scores. Brief screening that combines a resilience measure with an emotion regulation measure, alongside routine internalizing symptom assessment, may help detect this “silent” vulnerability during outpatient evaluations [23,24,54].

For this subgroup, preventive and skills-focused interventions may be particularly appropriate, including psychoeducation, problem-solving and coping skills training, and targeted support for social competence (e.g., structured social skills interventions or school-based counseling). Monitoring during predictable stress transitions (e.g., academic demands, peer stress, examination periods) may also be warranted in clinical follow-up, as low resilience may become clinically salient when environmental demands exceed available coping resources [21,23,24]. Future longitudinal and intervention studies are needed to directly examine whether early psychological or family-based interventions targeting emotion regulation and resilience can improve long-term adaptation in this subgroup. Importantly, these clinical considerations should be interpreted as hypothesis-generating rather than evidence-based treatment recommendations, given the exploratory and cross-sectional design of the present study. The findings do not establish causal pathways or intervention efficacy, but instead suggest directions for future longitudinal and interventional research. This interpretation should be considered provisional and requires confirmation in larger and preferably longitudinal samples to establish its stability and clinical significance.

Notably, differences in perceived competence were specific to the social domain, whereas academic and behavioral competence did not differ across profiles. This domain-specific pattern suggests that reduced resilience may be particularly salient in contexts requiring flexible interpersonal regulation rather than reflecting a generalized functional impairment [17,57].

### 4.3 Medication Patterns Across Emotional Profiles

Medication use differed systematically across emotional profiles. Methylphenidate treatment was more frequent in the Low Emotion Dysregulation / High Resilience / Low Internalizing profile, whereas SSRI use and internalizing comorbidity were most prevalent in the High Emotion Dysregulation / Low Resilience / High Internalizing profile. This co-occurrence is clinically expected and likely reflects indication-driven prescribing in naturalistic settings [58], whereby SSRIs are preferentially initiated when anxiety and depressive symptoms are prominent, rather than implying that SSRI treatment accounts for the emotional profile itself [59]. Accordingly, these findings should be interpreted descriptively.

Beyond its established effects on attention and behavioral control, accumulating evidence suggests that methylphenidate may also exert modulatory effects on emotional functioning in some individuals with ADHD [3,60]. Experimental and clinical studies indicate that stimulant treatment can reduce emotional lability [61,62], improve frustration tolerance [63], and attenuate affective reactivity [64], potentially through enhanced top-down regulatory control [11,65]. While the present cross-sectional design precludes conclusions regarding medication effects, the clustering of methylphenidate use within the Low Emotion Dysregulation / High Resilience / Low Internalizing profile is compatible with the possibility that effective stimulant treatment may contribute to emotional stabilization in a subset of adolescents [60,64]. However, given indication bias and unmeasured treatment history, these observations should not be interpreted as comparative effectiveness evidence.

Importantly, SSRIs should not be interpreted as first-line treatments for emotion regulation difficulties in ADHD, but rather as treatments initiated in response to clinically significant anxiety or depressive symptoms [59,66]. Conversely, while stimulant medications are primarily indicated for core ADHD symptoms, accumulating evidence suggests that they may indirectly support emotional regulation in some individuals [61,62,65], although this possibility cannot be evaluated within the present cross-sectional design.

Finally, the higher prevalence of SSRI use in the High Emotion Dysregulation / Low Resilience / High Internalizing profile underscores the clinical complexity of managing comorbid internalizing symptoms in ADHD [66,67,68]. These patterns highlight the importance of integrated treatment planning that considers emotional regulation capacities alongside symptom severity [67,69], rather than conceptualizing internalizing symptoms as secondary or unrelated concerns [70,71].

### 4.4 Parental Factors and Adolescent Autonomy

In the present study, parental self-reported emotion regulation difficulties and resilience did not differ across adolescent emotional profiles. Although parental emotion regulation and resilience were examined as correlates of the adolescent profiles, we did not include them in the clustering model to preserve a parsimonious adolescent-focused profile solution and to avoid conflating informant-level constructs. This finding is consistent with prior research indicating that associations between parental regulatory traits and adolescent emotional functioning may attenuate during adolescence, a developmental period marked by increasing emotional autonomy and a gradual shift away from family-centered regulation processes [28].

The existing literature on parental influences in ADHD similarly suggests stronger and more consistent associations in early childhood, with more heterogeneous and context-dependent findings emerging during adolescence [26]. One plausible explanation is that trait-level parental self-report measures may have limited sensitivity in capturing adolescent emotional heterogeneity in cross-sectional clinical samples, particularly when adolescents’ emotional functioning is increasingly shaped by extra-familial contexts such as peer relationships, school environments, and self-directed regulation strategies [24]. During this developmental stage, resilience may be more strongly influenced by peer connectedness, social acceptance, and perceived support outside the family, potentially weakening observable associations with parental trait-level characteristics assessed at a single time point [72].

In addition, participation-related selection bias cannot be excluded. Parents who consent to research participation and complete self-report measures during outpatient visits may represent a relatively more regulated or resourceful subgroup, thereby restricting variability in parental emotion regulation and resilience scores and further attenuating between-group differences [73,74]. Importantly, these findings should not be interpreted as evidence against family-level influences on adolescent emotional functioning. Rather, they suggest that adolescent emotional profiles may not map directly onto parental self-reported regulatory traits when assessed cross-sectionally. Longitudinal and multi-informant designs incorporating parental, peer, and contextual factors may be better suited to disentangle dynamic parent–adolescent regulatory processes and their evolving contributions to emotional resilience across development.

### 4.5 Limitations

Several limitations warrant consideration. First, the cross-sectional design precludes conclusions regarding temporal ordering or causal pathways, including those implied by mediation analyses, which should therefore be interpreted as a statistical decomposition of associations rather than evidence of directionality. Second, clustering solutions are inherently sample- and variable-dependent; alternative indicators, informants, or analytic approaches may yield different profile structures, underscoring the need for replication in independent samples. In addition, the exclusive use of a single clustering method (k-means) may limit the robustness of the identified profiles. Future studies employing alternative model-based approaches, such as latent profile analysis, would help to confirm cluster reproducibility. Third, reliance on adolescent self-reports for the core clustering variables may introduce shared-method variance, potentially inflating associations among emotion regulation difficulties, resilience, and internalizing symptoms. Fourth, although the sample size was adequate for exploratory clustering and mediation analyses, larger and more diverse samples are needed to enhance generalizability and to examine the stability of emotional profiles across developmental stages and clinical contexts. Fifth, parental emotion regulation and resilience were assessed using self-report measures in a volunteer clinical sample, which may have restricted variability due to participation-related selection effects and limited the detection of between-profile differences at the parent level. In addition, although basic family-level indicators such as parental education, family psychiatric history, and family structure were examined and did not differ significantly across clusters, broader socio-environmental factors (e.g., detailed socioeconomic indicators, family stress levels, school quality, and peer or social support) were not assessed in depth. These contextual dimensions may contribute meaningfully to emotional profile differentiation and external validity. Finally, medication use was assessed naturally and cross-sectionally; therefore, treatment effects cannot be disentangled from indication bias, illness severity, treatment duration, or prior treatment history. In addition, medication status was not included as a covariate in the clustering or mediation analyses, which may have influenced the observed profile differences and should be considered when interpreting these findings. Future studies should examine whether similar emotional profiles are observed in younger children and adults with ADHD, and whether profile structure varies across developmental stages. Although our sample was clinically referred, similar emotional configurations may plausibly be observed dimensionally in non-clinical or school-based populations, albeit with different prevalence and clinical salience. Replication in community samples is needed to evaluate external validity and the stability of the profile structure outside specialty care settings. The identified cluster structure may be partly dependent on the specific clustering algorithm and the selected variables, and thus should be interpreted as exploratory and subject to replication in independent samples.

## 5. Conclusions

Adolescents with ADHD exhibit distinct emotional profiles defined by differing configurations of emotion regulation difficulties, resilience, and internalizing symptoms. These profiles are clinically meaningful, relate to perceived social functioning, gender distribution, internalizing comorbidity, and medication patterns, and are largely independent of core ADHD symptom severity. Emotion regulation difficulties were statistically associated with the relationship between resilience and internalizing symptoms, highlighting their central role in adolescent emotional functioning.

Taken together, these findings support a shift from a uniform emotional risk model in ADHD toward profile-informed and developmentally sensitive assessment. Brief and routinely feasible self-report measures of emotion regulation and resilience may complement standard symptom-based evaluations and assist clinicians in identifying adolescents who require different emphases in clinical formulation and care planning. In particular, the identification of a Low-resilience silent profile underscores the importance of assessing adaptive capacity even when overt symptoms appear modest, as such adolescents may remain vulnerable under increasing developmental or contextual demands, while the highly vulnerable profile highlights the need for integrated interventions targeting both emotional regulation and internalizing symptoms alongside standard ADHD treatment.

## Data Availability

The data that support the findings of this study are available from the corresponding authors upon reasonable request.
