# Supplementary material for: Emotional Profiles in Adolescents With ADHD: A Person-Centered Analysis of Emotion Regulation, Resilience, and Internalizing Symptoms
Source: Alpha Psychiatry. 2026 Jul 31;27(4):50136. doi: 10.31083/AP50136 (PMC13540042; doi:10.31083/AP50136)
Supplement: Supplementary file 1 [file 2757-8038-27-4-50136-s1.zip › Supplementary Material.docx]

**Supplementary Table 1.** Subscale-Level Emotion Regulation and Internalizing Symptoms Across Emotional Clusters

Cluster sizes:
Cluster 1 (n = 39), Cluster 2 (n = 29), Cluster 3 (n = 19), Cluster 4 (n = 22)

**A. DERS Subscales**

| **Subscale** | **Cluster 1** | **Cluster 2** | **Cluster 3** | **Cluster 4** | **p value** |
| --- | --- | --- | --- | --- | --- |
| **Nonacceptance** | 4.54 ± 1.83 | 8.00 ± 2.88 | 5.00 ± 1.67 | 11.45 ± 2.92 | < .001 |
| **Goals** | 7.54 ± 2.17 | 10.34 ± 2.82 | 8.89 ± 2.56 | 13.14 ± 1.73 | < .001 |
| **Impulse** | 5.46 ± 2.16 | 8.62 ± 2.69 | 6.16 ± 2.19 | 11.91 ± 2.32 | < .001 |
| **Clarity** | 4.18 ± 1.73 | 5.66 ± 1.76 | 4.74 ± 1.15 | 6.27 ± 2.27 | < .001 |
| **Strategies** | 7.77 ± 2.10 | 13.00 ± 3.00 | 9.42 ± 3.17 | 18.73 ± 3.74 | < .001 |

**B. RCADS Subscales**

| **Subscale** | **Cluster 1** | **Cluster 2** | **Cluster 3** | **Cluster 4** | **p value** |
| --- | --- | --- | --- | --- | --- |
| **Social Phobia** | 6.82 ± 3.59 | 14.24 ± 5.19 | 13.84 ± 6.12 | 20.95 ± 3.68 | < .001 |
| **Panic Disorder** | 4.69 ± 3.64 | 8.97 ± 4.07 | 7.37 ± 5.72 | 17.45 ± 5.11 | < .001 |
| **Separation Anxiety** | 2.26 ± 2.06 | 5.41 ± 3.12 | 4.26 ± 3.44 | 8.32 ± 3.01 | < .001 |
| **Generalized Anxiety** | 5.03 ± 2.60 | 9.90 ± 3.38 | 7.89 ± 3.02 | 13.09 ± 3.12 | < .001 |
| **Obsessive–Compulsive** | 4.46 ± 3.29 | 7.69 ± 2.71 | 5.84 ± 3.45 | 11.09 ± 2.74 | < .001 |
| **Depression** | 6.77 ± 3.79 | 11.66 ± 4.09 | 10.63 ± 6.66 | 20.09 ± 4.52 | < .001 |

Values are presented as mean ± standard deviation for descriptive purposes.
Between-cluster comparisons were conducted using the Kruskal–Wallis test.
DERS: Difficulties in Emotion Regulation Scale; RCADS: Revised Child Anxiety and Depression Scale.

**Supplementary Table 2. Mediation Analysis:** Emotion Regulation Difficulties as a Mediator Between Resilience and Internalizing Symptoms

**Model specification
X:** Resilience (CD-RISC-25 total) **M:** Emotion regulation difficulties (DERS-16 total) **Y:** Internalizing symptoms (RCADS internalizing total) **Bootstrap resampling: 5,000 (bias-corrected, 95% CI)**

**Path coefficients**

| **Path / Effect** | **b** | **SE** | **t** | **p** | **95% CI** |
| --- | --- | --- | --- | --- | --- |
| **a: Resilience → Emotion dysregulation** | **−0.26** | **0.08** | **−3.32** | **.001** | **—** |
| **b: Emotion dysregulation → Internalizing (controlling for resilience)** | **1.20** | **0.13** | **9.58** | **< .001** | **—** |
| **c′: Resilience → Internalizing (direct effect)** | **−0.28** | **0.11** | **−2.61** | **.010** | **—** |
| **c: Resilience → Internalizing (total effect)** | **−0.60** | **0.14** | **−4.28** | **< .001** | **—** |

**Indirect effect**

| **Effect** | **b** | **BootSE** | **95% CI** |
| --- | --- | --- | --- |
| **Indirect effect (a × b)** | **−0.32** | **0.11** | **[−0.54, −0.11]** |

**Model summary statistics**

| **Outcome variable** | **R²** | **F** | **df** | **p** |
| --- | --- | --- | --- | --- |
| **Emotion dysregulation (M)** | **0.094** | **11.0** | **1, 107** | **.001** |
| **Internalizing symptoms (Y)** | **0.542** | **62.4** | **2, 106** | **< .001** |

**Table notes**

Unstandardized coefficients (b) are reported.
Bootstrap standard errors (BootSE) and 95% confidence intervals are reported, with inference based primarily on the bootstrap confidence intervals.
No covariates were included in the mediation model.
CD-RISC: Connor–Davidson Resilience Scale; DERS: Difficulties in Emotion Regulation Scale; RCADS: Revised Child Anxiety and Depression Scale.

**Supplementary Fig. 1.** Six-cluster (k = 6) solution illustrating profile fragmentation

Radar plot depicting standardized mean levels of emotion dysregulation, resilience, and internalizing symptoms for the six-cluster (k = 6) k-means solution. Values are presented as z-scores to facilitate comparison across dimensions. Although the six-cluster solution received quantitative support during model evaluation, visual inspection suggests substantial overlap in profile geometry, with clusters differing primarily in magnitude rather than qualitative pattern. This fragmentation without added interpretive clarity contrasts with the more distinct configurations observed in the four-cluster solution presented in the main text (Figure 3) and supports selection of the more parsimonious four-cluster model as the final solution.

**Supplementary Fig. 2.** Stability and model evaluation across candidate cluster solutions (k = 2–12).

**Panel A.** Mean Davies–Bouldin Index (DBI), Silhouette coefficient, and Linear Discriminant Analysis (LDA) separability accuracy across candidate cluster solutions (k = 2–12). Values represent means ± standard deviation across repeated random initializations. Lower DBI values and higher Silhouette and LDA accuracy values indicate better clustering performance and separability. Mid-range solutions (k = 4–6) demonstrated favorable and relatively stable performance across metrics. LDA separability accuracy refers to the classification accuracy obtained when predicting cluster membership using linear discriminant analysis with cross-validation.

**Panel B.** K-means inertia (sum of squared errors, SSE) and composite ranking score across candidate cluster solutions. The dashed vertical line indicates the elbow region identified by inspection of the inertia curve (k ≈ 5). The composite ranking integrates normalized clustering performance metrics and their across-run stability. The four-cluster solution demonstrated competitive ranking performance while maintaining parsimony.

Together, these results support the quantitative adequacy and relative stability of mid-range cluster solutions within the present sample, while also illustrating the absence of a single unequivocal optimum across all indices.

**Supplementary Fig. 3.** Stability of cluster solutions across random initializations and optimization settings.

**Panel A.** Mean pairwise Jaccard similarity across repeated k-means runs (n_init = 1) for candidate cluster solutions (k = 2–12). Values represent mean ± 1 standard deviation across repeated runs. As expected, stability gradually decreased with increasing k, reflecting greater fragmentation and sensitivity to initialization at higher cluster numbers. Moderate stability was observed for mid-range solutions, including k = 4.

**Panel B.** Mean pairwise Jaccard similarity across repeated runs under extensive internal optimization (n_init = 100). Under this setting, solutions up to k = 6 demonstrated near-perfect convergence (mean Jaccard ≥ 0.97), indicating that the algorithm consistently converged to highly similar partitions across repeated optimizations. Stability decreased as the number of clusters increased beyond four, suggesting reduced robustness of higher cluster solutions.
